# Prevalence of *IRF4* rearrangement in large B-cell lymphomas of the Waldeyer’s ring in adults

**DOI:** 10.1007/s00428-023-03516-7

**Published:** 2023-02-22

**Authors:** Sebastian Streich, Leonie Frauenfeld, Franziska Otto, Barbara Mankel, Irina Bonzheim, Falko Fend, Leticia Quintanilla-Martinez

**Affiliations:** Institute of Pathology and Neuropathology, Eberhard Karls University of Tübingen and Comprehensive Cancer Center, University Hospital Tübingen, Liebermeisterstrasse 8, 72076 Tübingen, Germany

**Keywords:** Diffuse large B-cell lymphoma, Waldeyer’s ring, *IRF4* rearrangement, *IRF4* mutations

## Abstract

Diffuse large B-cell lymphoma (DLBCL) is the most common B-cell non-Hodgkin lymphoma (B-NHL) in adults. These lymphomas are classified according to gene expression profiling (GEP) into germinal center B-cell (GCB) and activated B-cell type (ABC). Recent studies have suggested new subtypes of large B-cell lymphoma, based on genetic and molecular alterations, among them is large B-cell lymphoma with *IRF4*-rearrangement (LBCL-*IRF4*). We used fluorescence in situ hybridization (FISH), GEP (using the DLBCL COO assay by HTG Molecular Inc), and next generation sequencing (NGS) to comprehensively characterize 30 cases of LBCLs located in Waldeyer’s ring in adult patients and to identify LBCL*-IRF4*. FISH revealed breaks of *IRF4* in 2/30 cases (6.7%), *BCL2* breaks in 6/30 cases (20.0%), and IGH breaks in 13/29 cases (44.8%). GEP classified 14 cases each as GCB or ABC subtype, and 2 cases remained unclassified; this was concordant with the immunohistochemistry (IHC) in 25/30 cases (83.3%). A subgrouping, based on GEP, was performed: group 1 included 14 GCB cases with the most frequent mutations in *BCL2* and *EZH2* in 6/14 cases (42.8%). The two cases with *IRF4* rearrangement were assigned to this group by GEP and showed *IRF4* mutations, supporting the diagnosis of LBCL-*IRF4*. Group 2 included 14 ABC cases; the most frequent mutations were *CD79B* and *MYD88* identified in 5/14 patients (35.7%). Group 3 included 2 unclassifiable cases in which no molecular patterns were detected. Overall, LBCLs of Waldeyer’s ring in adult patients are a heterogeneous group, including LBCL-*IRF4*, which shares several features with cases in the pediatric population.

**Supplementary Information:**

The online version contains supplementary material available at 10.1007/s00428-023-03516-7.

## Introduction

Diffuse large B-cell lymphoma (DLBCL) is the most common B-cell non-Hodgkin lymphoma (B-NHL) in adults. DLBCL are classified based on gene expression profiling (GEP) into germinal center B-cell-type (GCB), activated B-cell-type (ABC), and an unclassifiable subgroup, according to the putative cell of origin (COO). As a surrogate marker, immunohistochemistry (IHC) is currently used in routine diagnosis. Although there are different immunohistochemical algorithms, the Hans algorithm (HA) is the most widely used [1–3]. HA divides DLBCL into GCB and non-GCB based on three antibodies, namely CD10, BCL6, and MUM1 [2]. However, the prognostic impact of IHC algorithms is limited. Trials in DLBCL using COO based on IHC analysis has given disappointing results [4, 5]. More recently, a genetic classification based on structural variants, mutational profile, and somatic copy-number alterations (CNA) was proposed. This genetic classification can further dissect and stratify the GEP-based COO classification into seven genetic subtypes with putative clinical relevance [6, 7]. Hans algorithm remains an important diagnostic tool in routine diagnosis; however, mutational profile of DLBCL contributes to understand the pathogenesis of DLBCL and has the potential to direct targeted therapy in the future [8]. Nevertheless, these mutational subgroups need to be validated and have still only limited relevance for routine diagnostics and treatment. The distinction between ABC and GCB, although it does not capture the complexity of DLBCL, reflects a basic biological distinction, and moving forward, a combination of COO and molecular subgroups may provide a better stratification of the disease [9, 10].

The 2017 World Health Organization (WHO) classification of lymphoid neoplasms introduced for the first time the large B-cell lymphoma with *IRF4* rearrangement (LBCL-*IRF4*) as a provisional entity [11]. This disease is now recognized as definitive entity in both the International Consensus Classification [9] and in the 5th edition of the WHO [12]. LBCL-*IRF4* predominates in children and young adults, shows frequent involvement of the Waldeyer’s ring (WR), and has an excellent outcome with chemotherapy. Morphologically, it is composed of large cells, and the growth pattern might be diffuse, follicular/diffuse, or purely follicular [13]. LBCL-*IRF4* in children and young adults has a distinct molecular profile characterized by frequent mutations of *IRF4*, as a result of aberrant somatic hypermutation (aSHM), and NF-κB-related genes despite a GCB-transcriptional program. LBCL-*IRF4* has been reported also in adults. However, adult cases show higher genetic complexity, higher mutational burden with frequent *MYD88* and *KMT2D* mutations, and more often an ABC-GEP [14].

Although LBCL-*IRF4* in children and young adults are frequently reported in the Waldeyer’s ring, this association has not been thoroughly investigated in adults. Interestingly, a previous study in an adult population suggested that DLBCL of Waldeyer’s ring has distinctive clinicopathological features with high rate of GCB phenotype (61%) and a variable follicular pattern, observed in 44% of the cases [15]. The aim of this study was to comprehensively analyze the genetic landscape of DLBCL and follicular lymphoma (FL) grade 3B of the Waldeyer’s ring in adult patients and to investigate the frequency of LBCL-*IRF4* by fluorescence in situ hybridization (FISH), NGS, and GEP.

## Material and methods

### Patient samples

Thirty samples of adult patients, diagnosed as DLBCL or FL grade 3B of the Waldeyer’s ring (includes lymphoid tissues in the nasopharynx, palatine tonsils, tongue base, soft palate, and oropharyngeal wall) between 2002 and 2020, were retrieved from the files of the Institute of Pathology, University Hospital Tübingen. The cases were classified following the criteria of the 2017 WHO classification [16]. The immunohistochemical staining was performed as part of the diagnostic workup. Expression of the immunohistochemical marker in more than 30% of the neoplastic cells was considered positive. The study was conducted in accordance with the Declaration of *Helsinki* and was approved by the local Ethics Review Committee and the IRB review panels of the contributing institutions (UKT 199/2020/BO2).

### *Fluorescence *in situ* hybridization (FISH)*

FISH analyses were performed using 3.5 µm thick FFPE sections and the following probes: *IRF4/DUSP22* Dual Color Break Apart Probe (ZytoVision GmbH, Bremerhaven, Germany), IGH Dual Color Break Apart Probe (ZytoVision GmbH), *BCL2* Dual Color Break Apart Rearrangement Probe (Abbott Molecular, Des Plaines, IL, USA), and *MYC* Break Apart Rearrangement Probe (Abbott Molecular). The analyses were carried out according to the manufacturer’s protocols. Results were considered positive if the percentage of positive cells was greater than or equal to 10%.

### Gene expression profiling (GEP)

GEP analysis was performed using the EdgeSeq system (HTG Molecular Diagnostics Inc., Tucson, AZ, USA). All samples were analyzed using the HTG EdgeSeq DLBCL Cell of Origin assay for Ion Torrent platforms (HTG Molecular Diagnostics Inc.), according to the manufacturer’s protocol [14]. The COO classification was performed using the HTG EdgeSeq Parser. For the statistical analyses, a quantile normalization, according to Bolstad et al. [17], was performed (see also section statistical analysis). Further details on the GEP methodology can be found in the supplemental methods, gene expression profiling section.

### Next-generation sequencing (NGS)

NGS panel sequencing was performed using the Ion GeneStudio S5 prime (Thermo Fisher Scientific, Waltham, MA, USA) with an AmpliSeq Custom DLBCL Panel, designed using the Ion AmpliSeq Designer (Thermo Fisher Scientific). All cases were screened for mutations with a customized panel, encompassing *BCL2*, *BCL6*, *CARD11*, *CD79B*, *EZH2*, *IRF4*, *MYD88*, *PIM1*, *PRDM1*, and *TNFAIP3*. For *CD79B*, *EZH2* and *MYD88* hotspot regions are covered (supplemental table 1). In addition, single amplicons were created for some gene regions, including *IRF4* (see also supplemental table 2). The analyses performed for each case are shown in supplemental table 3. Detailed information on the next-generation sequencing is shown in the supplemental methods, mutational analysis section.

Cases with mutations in *CD79B* and/or *MYD88* genes were considered to belong most probably to MCD/C5 subtype, whereas *EZH2* mutations and/or *BCL2* translocations were considered to belong most probably to EZB/C3 subtype [6, 7].

### Statistical analysis

All statistical tests were two-sided, and statistical significance was concluded for values *p* < 0.05. GEP data was quantile normalized. Significance tests were performed using the Mann–Whitney *U* test and the Kruskal–Wallis H test. Statistical analyses were carried out using RStudio, version 1.2.1335, and the IBM SPSS Statistics software, version 28 for Microsoft Windows.

## Results

### Clinicopathological features

Clinical information is summarized in Table 1. A total of 30 patients were included in the study, of whom 12 were male and 18 were female patients, representing an M:F ratio of 1:1.5 with a median age at initial presentation of 69.5 years (range 37–87 years). Information about the clinical stage was available in 23/30 cases, of which 18 had low clinical stage (I or II) at presentation (18/23, 78%), whereas five patients (5/23, 22%) had high-stage disease (III or IV). IPI score was available for 20/30 patients and was low in most cases (14/20, 47%), low-intermediate in three cases (3/20, 10%), high-intermediate in two cases (2/20, 7%), and high in one case (1/20, 3%). Nineteen out of 23 patients (83%) received therapies containing R-CHOP, including 2 patients with a reduced dose of CHOP, whereas three patients (3/23, 13%) received other therapies. One patient (1/23) received no therapy. Clinical follow-up was obtained in 22/30 patients with a mean of 55.0 months (range 1–125 months). Complete remission (CR) was achieved in 17/18 patients, including one patient (case no. 21) who had a relapse (REL) at 69 months of follow-up, but this patient achieved CR with renewed therapy, leading to sustained CR after a total of 109 months of follow-up. One patient achieved partial remission (PR).

**Table 1:** Clinical data of 30 large B-cell lymphomas of Waldeyer’s ring in adults

Case #	Sex	Age	Diagnosis	Clinical stage	IPI	Treatment	R/R	Follow-up
1	F	44	DLBCL	NA	NA	NA	NA	NA
2	F	45	DLBCL	IIA	Low	6 × R-CHOP	CR	125 mo NED
5	F	81	FL 3B	NA	NA	NA	NA	NA
6	F	64	DLBCL	II	Low-inter	MAIN 6 × R-CHOP 6 × R	CR	61 mo NED
7	F	79	DLBCL	NA	NA	NA	NA	NA
8	F	57	DLBCL	IIA/E	Low	6 × R-CHOP	CR	120 mo NED
9	F	70	FL 3B	IIE	Low	6 × R-CHOP	CR	98 mo NED
10	F	69	DLBCL	IIA/E	Low	1 × R-CHOP	NA	NA
11	M	52	DLBCL	II	Low	6 × R-CHOP	CR	84 mo NED
13 §	F	78	DLBCL	II	Low	4 × R-CHOP	CR	97 mo NED
15	M	47	DLBCL	IA	Low	4 × R-CHOP 2 × R	CR	72 mo NED
16	M	78	DLBCL	IIA	Low	4 × R-CHOP 4 × R	CR	53 mo NED
17	F	79	DLBCL	IIA	Low	6 × R-CHOP 2 × R	CR	58 mo NED
19	F	87	DLBCL	IV	NA	No treatment	NA	1 mo DOD
20	F	78	DLBCL	NA	NA	NA	NA	NA
21	M	52	DLBCL	III	Low-inter	8 × R-CHOP 8 × R R-BEAM SCT	REL CR	REL after 69 mo, NED after a total of 109 mo
22	F	47	DLBCL	IIA	Low	4 × R-CHOP RTX 39,6 Gy	CR	45 mo NED
23	F	74	DLBCL	IVB/E	High	4 × R-B	PR	5 mo LFU
24	M	74	DLBCL	IIA/E	Low	6 × R-CHOP	CR	65 mo NED
25	F	63	DLBCL	IA	High-inter	6 × R-CHOP 2 × R MTX	CR	48 mo NED
26	F	70	DLBCL	NA	NA	NA	NA	NA
27	M	62	DLBCL/ FL 3B	IIA	NA	4 × R-CHLiP 4 × R	CR	48 mo NED
29	M	81	DLBCL	IIIA	Low-inter	1 × R-miniCHOP	NA	NA
30	M	71	DLBCL	NA	NA	NA	NA	2 mo LFU
31	F	82	DLBCL	IA	NA	3 × R-miniCHOP	NA	2 mo LFU
32	M	61	DLBCL	IVA	High-inter	TEAM SCT	NA	18 mo LFU
33	M	58	DLBCL	I	Low	4 × R-CHOP 2 × R	CR	17 mo NED
34	M	37	DLBCL	IIA	Low	6 × R-CHOP 2 × R	CR	51 mo NED
35^§^	F	37	DLBCL	IA	Low	6 × R-CHOP 2 × R	CR	30 mo NED
36	M	77	DLBCL	NA	NA	NA	NA	NA

*M*, male; *F,* female; *IPI*, International Prognostic Index; *NA*, not available; mo, months; *LFU*, lost to follow-up; *R/R*, relapse/refractory; *NED*, no evidence of disease; *DOD,* died of disease; *PR*, partial remission; *CR*, complete remission; *REL*, relapse. Therapy: MAIN: study treatment with R-CHOP with/without Bevacizumab (NCT00486759); *MTX*, methotrexate; *R*, rituximab; *R-B*, rituximab, bendamustine; *R-BEAM*, rituximab, carmustine, etoposide, cytarabine, melphalan; *R-CHLiP*, rituximab, cyclophosphamide, liposomal vincristine, prednisone; *R-CHOP*, rituximab, cyclophosphamide, doxorubicin, vincristine, prednisone; *R-miniCHOP*, rituximab with dose-reduced CHOP (see above); *RTX*, radiotherapy; *SCT*, stem cell transplantation; *TEAM*, thiotepa, etoposide, cytarabine, melphalan

^§^Cases with IRF4 translocation/mutation

**Fig. 1:**
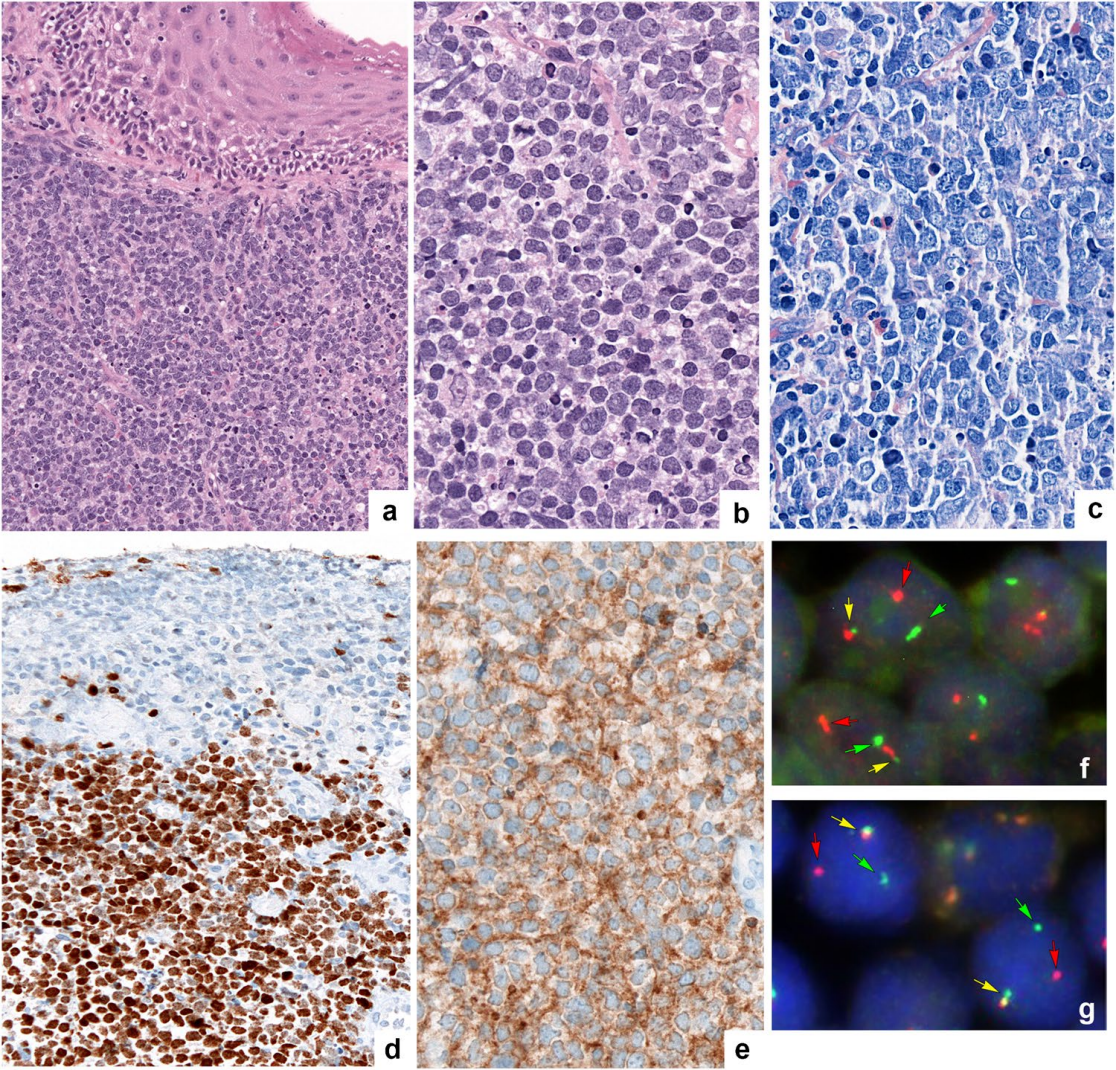
Morphological, immunohistochemical, and genetic features of LBCL-*IRF4* (case no. 35). **a** Large B-cell lymphoma with blastoid morphology with medium-sized cells, inconspicuous nucleoli, and scant cytoplasm (original magnification × 200; hematoxylin and eosin stain); **b** with original magnification × 400: hematoxylin and eosin stain; **c** original magnification × 400: Giemsa stain. **d** MUM1. **e** CD10. **f** Fluorescence in situ hybridization (FISH) analysis using a break apart probe (BAP) demonstrates an *IRF4* break with 1 colocalized signal (yellow arrow) and 1 split signal (green and red arrows) consistent with gene rearrangement. **g** FISH analysis with a BAP demonstrates an IGH break with 1 colocalized signal (yellow arrow) and 1 split signal (green and red arrows) consistent with gene rearrangement (Fig. 1 was created using Adobe Photoshop Version CS6)

Morphologically, 28 cases were diagnosed as DLBCL, NOS and two as FL grade 3B. One case showed a blastoid morphology with medium-sized cells, inconspicuous nucleoli, and scant cytoplasm (Fig. 1a–c). The rest of the cases showed a characteristic centroblastic morphology. According to the Hans algorithm, 18/30 (60%) cases were classified as GCB type, including five cases, which were CD10 + , BCL6 + , MUM1 + (Fig. 1d, e), whereas 12/30 cases (40%) were classified as non-GCB type. CD10 was positive in 16/30 (53%) cases, BCL6 in 28/30 (93%), and MUM1 in 17/30 (57%). BCL2 expression was observed in 23/30 (77%) cases, whereas MYC was expressed in only 4/27 (15%) cases. The proliferation rate, measured by MIB1, was high in nearly all cases (> 60% in 27/30 cases).

No significant age difference could be shown between DLBCL ABC (mean age 68.8 years/median age 74 years, *SD* = 13.6, range 45 to 87 years) and DLBCL GCB (mean age 63.2 years/median age 66.5 years, *SD* = 13.3, range 37 to 79 years) (*p* = 0.194).

### *Fluorescence *in situ* hybridization*

FISH analyses for *IRF4*, *BCL2*, and IGH were performed in all cases. *MYC* analysis was done in cases with MYC expression ≥ 40% of the tumor cells. FISH analysis demonstrated a break of *IRF4*, indicative of a translocation, in 2/30 cases (6.7%), a *BCL2* break in 6/30 cases (20%), and an IGH break in 13/29 cases (44.8%). No *MYC* break was identified in the 4 cases analyzed. Both cases with an *IRF4* break also showed an IGH break (Fig. 1f, g), whereas only in four cases (4/6; 66%) with a *BCL2* break, a concordant IGH break was demonstrated. In seven cases, a matching partner for the IGH break was not identified, but *BCL6* FISH was not performed. Additionally, *IRF4* gains were detected in seven cases (23.3%), *BCL2* gains in two cases (6.7%), and IGH gains in one case (3.4%), whereas no *MYC* gains were detected.

### Mutational analyses

**Fig. 2:**
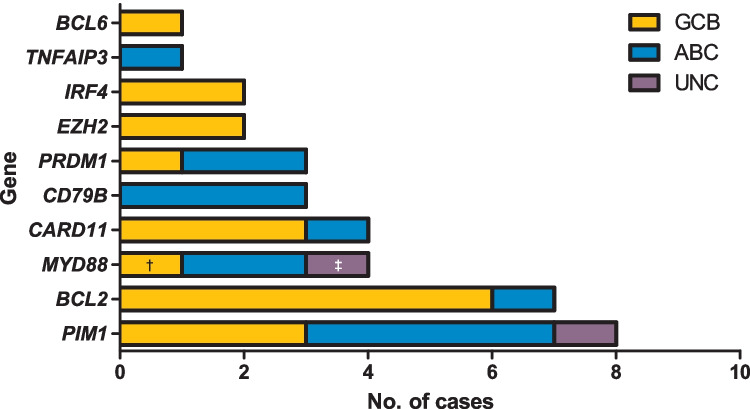
Mutation frequencies across GEP subtypes. Total number of cases (x-axis) with a mutation in the gene depicted on the y-axis. ^†^Case no. 23 was classified as GCB by GEP and showed a non-canonical mutation of *MYD88* in position p.S219C. ^‡^Case no. 10 was unclassifiable by GEP and showed a non-canonical mutation of *MYD88* in position p.S243N (Fig. 2 was created using GraphPad Prism version 5.00)

All cases were analyzed by NGS. The results are summarized in Fig. 2. Sixty-five variants were identified in 20/30 (67%) cases (supplemental table 4). Ten cases (33%) were wild type for all analyzed genes. The mean mutational burden was 2.2 variants per case. Overall, the allelic frequency of the mutations ranged from 7 to 89% with a mean of 33.6% (*SD* = 19.1). Among the mutated cases, 10 of 30 cases showed only one mutated gene (33%), five cases demonstrated two mutated genes (5/30, 25%), and five cases revealed three mutated genes (5/30, 25%). Overall, 58 missense, 3 nonsense, 2 frameshift, and 2 splice site mutations were found. The most frequent genetic alterations were found in *PIM1* with 8 mutated cases (8/30, 27%), followed by *BCL2* with seven mutated cases (6 of them with *BCL2*-rearrangement) (7/30, 23%), *CARD11* and *MYD88* with four mutated cases each (4/30, 13%), *CD79B* and *PRDM1* with three mutated cases each (3/30, 10%), *IRF4* and *EZH2* with two mutations each (2/30, 6.7%), and *BCL6* and *TNFAIP3* with one mutated case each (1/30, 3%).

### Gene expression profiling

**Fig. 3:**
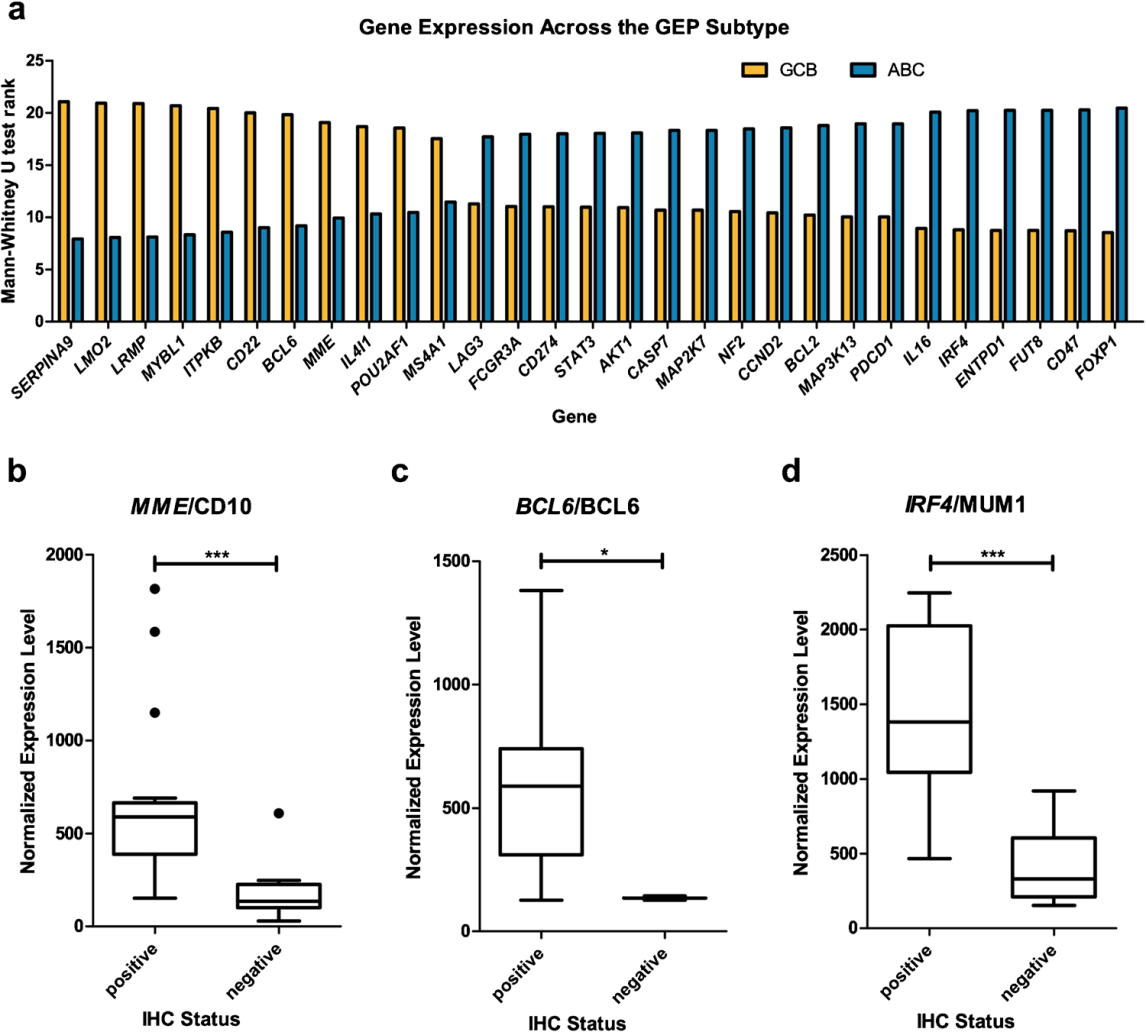
Results of the gene expression profiling. **a** Genes with significant (*p* < 0.05) differences in the expression levels across GEP subtypes. In total, 11 genes were highly expressed in the GCB subgroup and 18 ABC subgroup respectively. **b**–**d** Correlation of the immunohistochemical status (x-axis) with the normalized expression level of the GEP (y-axis) for *MME*/CD10 (**b**), *BCL6*/BCL6 (**c**), and *IRF4*/MUM1 (**d**). The differences were significant with *p* < 0.001 for *MME*/CD10, *p* = 0.018 for *BCL6*/BCL6, and *p* < 0.001 for *IRF4*/MUM1 (Fig. 3 was created using GraphPad Prism version 5.00)

COO classification, using the HTG EdgeSeq Parser, classified 14 cases each as GCB and ABC, whereas two cases remained unclassified (UNC). Significant differences in gene expression among the subtypes were detected in 29 of the gene products examined; 11 of them were highly expressed in the GCB subtype, whereas 18 were highly expressed in the ABC subtype (Fig. 3a and Supplemental Fig. 1). The correlation between the protein and mRNA expression for CD10/*MME*, BCL6/*BCL6*, and MUM1/*IRF4* was excellent (Fig. 3b–d).

### Correlation of morphological features, FISH, GEP, and mutational analysis

**Fig. 4:**
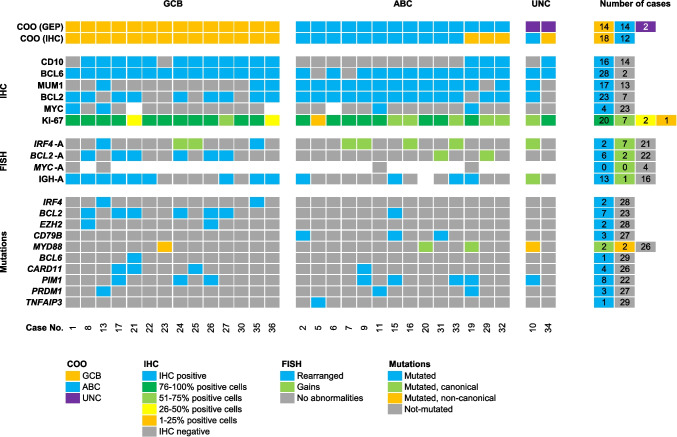
Overview of 30 cases of LBCL-*IRF4* clustered in groups according to the COO (GEP). This overview includes the COO results by GEP and IHC, IHC results, and FISH status and mutations. While each column represents one case, each line represents a specific analysis. On the right side of the figure, the raw numbers of specific analyses are shown (Fig. 4 was created using Microsoft Excel version 2210)

The correlation of all parameters is depicted in Fig. 4. The agreement between the COO classification using GEP and IHC was 83% (25/30 cases). From the five discordant cases using GEP as the gold standard, three were in the ABC subtype and two in the unclassifiable cases. The 30 cases were assigned according to GEP into three different groups:

1. Group 1: 14 cases were classified as GCB type by GEP and IHC. All cases expressed CD10 (12/14 cases, 86%) and/or BCL6 (14/14 cases, 100%) with 3 cases expressing MUM1/*IRF4* (3/14 cases, 21%). *BCL2*-R was demonstrated in 6 cases (49%) and *IRF4*-R in 2 cases (14%). All of the *BCL2* rearranged cases showed *BCL2* mutations. Mutational analysis showed that the most frequent mutations were *BCL2/EZH2* found in 6 cases (43%). Other mutations frequently found in this group were *CARD11* and *PIM1* (21.4%, each). This group includes the two *IRF4* rearranged cases that were the only cases with *IRF4* mutations. One case showed an uncommon immunohistochemical and genetic signature (case no. 23). This case was classified as GCB by GEP and IHC while lacking the expression of CD10 and carrying a non-canonical *MYD88* mutation (p.S219C).
2. Group 2: 14 cases were classified as ABC type by GEP and 11 of these cases by IHC. The three discrepant cases were assigned into the GCB group according to the Hans algorithm because of CD10 expression. By FISH analysis, no *BCL2*, *MYC*, or *IRF4* translocations were identified. The most frequent mutations were *CD79B* and *MYD88* identified in 5 patients (36%). Other mutations identified were *PIM1* (26%) and *PRDM1* (14%). One case of this group (case no. 15) was classified as ABC by GEP and IHC and showed mutations of *BCL2*, *CD79B*, and *PIM1*. Furthermore, this case showed a rearrangement of IGH while lacking demonstrable rearrangements in other genes. Because of the *BCL2* mutations and an IGH rearrangement without identifiable partner, the possibility of a cryptic *BCL2* translocation cannot be excluded.
3. Group 3: 2 cases were unclassifiable by GEP; however, by Hans algorithm, one case each was assigned to the GCB and the non-GCB group. The case with CD10 and BCL6 expression (no. 34) showed no translocations or mutations. The case with MUM1/*IRF4* expression (no. 10) had an *MYD88* and *PIM1* mutation supporting the non-GCB-type suggested by the Hans algorithm, although the *MYD88* mutation was not in the hotspot region.

## Discussion

We investigated the morphological and genetic features of DLBCLs and FLs grade 3B of Waldeyer’s ring in 30 adult patients and screened these cases by FISH analysis for *IRF4* translocations to identify cases of the novel entity LBCL-*IRF4*. Classification of these cases according to their COO using IHC and GEP revealed equal numbers of DLBCL of GCB-type (group 1: 47%) and ABC-type (group 2: 47%). Mutational analysis supported the GCB-type (*BCL2/EZH2* mutations) in 43% of the cases and the ABC-type (*MYD88*/*CD79B* mutations) in 36% of the cases. Our study demonstrated that LBCL-*IRF4* in adults occurs in the WR in 7% of the cases.

In this study, DLBCL of GCB-type was slightly below the rate reported in the WR in a previous study (46.7 vs 62%) [15]. However other groups have found even lower frequency of GCB-type DLBCL (20.4%) [18]. In general, unselected studies of extranodal and nodal DLBCL have reported GCB rates between 40 and 47% [2, 19]. COO assignment was possible in all examined cases in this cohort using the HA by IHC. GEP was able to assign 93% of all cases examined. The agreement between IHC and GEP was over 80%. An important source of disagreement between IHC and GEP is the aberrant expression of CD10 + BCL6 + MUM1 + demonstrated in five cases. These cases per default are classified as GCB type due to CD10 expression. Two of these latter cases were assigned by GEP into the ABC group with gene mutation characteristic of the ABC type. These results support the conclusion that cases with CD10 + , BCL6 + , and MUM1 + should not be classified by HA [14].

DLBCL assigned to COO GCB-type by GEP expressed either CD10 and/or BCL6 (group 1). Only 2 cases did not express CD10. Importantly, three cases in this group coexpressed MUM1/IRF4, thus showing an aberrant phenotype, two of them with *IRF4*-rearrangement. This finding indicates that cases with aberrant expression of CD10 and MUM1/IRF4 should be investigated for *IRF4*-rearrangements. Both cases showed a mutation in *IRF4*, most probably due to aberrant somatic hypermutation. *BCL2* rearrangements were demonstrated in half of the cases, sometimes with coexisting *BCL2*-mutations. The rate of *BCL2* translocations in our cohort was more than twice as high as in other studies investigating WR DLBCLs, depending on the comparison reference source, but matched very well with results from studies that included both nodal and extranodal LBCLs [15, 18, 20]. In the majority of the *BCL2*-rearranged cases, an additional IGH translocation was demonstrated, suggesting the characteristic *BCL2*:IGH translocation. In two *BCL2*-rearranged cases, IGH break was not documented indicating the possibility of alternative translocation partners with the light immunoglobulin chain genes κ and λ [21].

According to GEP, 14 DLBCL were assigned to the ABC group (group 2). Eleven of 14 cases showed concordance between HA and GEP with CD10-negativity. In contrast, 3/14 cases, despite the expression of CD10, were assigned to the ABC group based on the GEP and mutational landscape. Two of these latter cases showed aberrant coexpression of CD10, BCL6, and MUM1. No *IRF4* translocations were identified in this group. The most frequent mutations (7/14 cases, 50.0%) involved genes of the NF-κB pathway (*CD79B*, *MYD88*, *CARD11*, *TNFAIP3*) consistent with the MCD/CD5 molecular group [6, 7]. In the study of Chapuy et al., cohort mutations in *CD79B* and *MYD88* were identified in 48% and 50%, respectively [7]. The aberrant activation of NF-κB is well described in the ABC-type and contributes to the pathogenesis of DLBCL, opening up possibilities for targeted anti-NF-κB therapy. Interestingly, two recent studies of LBCL-*IRF4* in pediatric and adult populations also demonstrated enrichment in mutations affecting the NF-κB pathway (*CARD11*, *CD79B*, *MYD88*, *TNFAIP3*) [14, 22].

Finally, one of the aims of this study was to investigate the prevalence of LBCL-*IRF4* in the WR in adult patients. Our cohort shows similar results to other adult patients cohorts in and outside Waldeyer’s ring, with a frequency of 8% [15] and in a mixed cohort of children and adults with 13% [13]. Most of the *IRF4*-rearranged cases in the study by Salaverria et al. [13] affected children and young adults under the age of 18 (14/23 cases). However, studies including only children and young adults have reported higher frequencies, between 21 to 32% of *IRF4* rearranged cases, mainly involving the WR [23, 24]. Only one study in adults has reported a relative high frequency (24.5%) of LBCL-*IRF4* in the WR in a Chinese population [18]. The mean age of the Chinese cohort was lower when compared to our cohort (58 years vs 69 years); however, this does not explain the difference. The two identified cases with *IRF4* rearrangement in our cohort showed both IGH rearrangements, a triple positive phenotype (CD10 + , BCL6 + , MUM1 +), and GCB-type by GEP. The patients were 78 and 37 years old at time of diagnosis, and both showed limited disease (clinical stage II and IA, IPI low); both were treated with R-CHOP and showed no evidence of disease with complete remission at 97 and 30 months. As has been previously reported, mutations in *IRF4* were detected only in the two cases with *IRF4*-translocations and showed the pattern of aberrant somatic hypermutation [14, 22]. Thus, reinforcing the contention that the presence of *IRF4* mutations, in the correct context, is supportive of the diagnosis of LBCL-*IRF4*. Although this lymphoma is more frequently observed in children and young adults, cases in adults share the GCB-type, *IRF4* mutations, and frequent coexpression of CD10, BCL6, and MUM1 [22].

In conclusion, large B cell lymphomas involving the Waldeyer’s ring were equally divided in GCB and ABC type. The overall concordance in this study between GEP and HA was excellent except for those cases expressing aberrantly CD10, BCL6, and MUM1. LBCL-*IRF4* in adult patients in this study share clinical, morphologic, and genetic features with LBCL-*IRF4* in pediatric populations. This relatively small cohort in adults suggests a lower prevalence of LBCL-*IRF4* in the Waldeyer’s ring in adults compared to pediatric population. This finding needs to be confirmed in a larger cohort.

## Supplementary Information

Below is the link to the electronic supplementary material.

Supplementary file1 (PDF 468 KB)

## Data Availability

The datasets generated during the current study are available from the corresponding author on reasonable request.
