# Supplementary material for: Prevalence of IRF4 rearrangement in large B-cell lymphomas of the Waldeyer’s ring in adults
Source: Virchows Arch. 2023 Feb 22;482(3):551–60. doi: 10.1007/s00428-023-03516-7 (PMC10033557; doi:10.1007/s00428-023-03516-7)
Supplement: Supplementary file 1 — Supplementary file1 (PDF 468 KB) [file 428_2023_3516_MOESM1_ESM.pdf]

Supplemental Material

# Prevalence of *IRF4* Rearrangement in Large B-Cell Lymphomas of the Waldeyer's Ring in Adults

Sebastian Streich, Leonie Frauenfeld, Franziska Otto, Barbara Mankel, Irina Bonzheim, Falko Fend, Leticia Quintanilla-Martinez

# Contents

Supplemental methods .......... 3
Gene expression profiling (GEP) .......... 3
Mutational analysis – targeted Next Generation Sequencing .......... 3
Supplemental tables .......... 5
Supplemental figures .......... 16
Supplemental references .......... 17

# Supplemental methods

## Gene expression profiling (GEP)

Next generation sequencing (NGS-)based gene expression profiling (GEP) was performed to classify the DLBCL samples into GCB, ABC or unclassifiable subtypes using the HTG EdgeSeq System (HTG Molecular Diagnostics Inc., Tucson, AZ, USA). HTG gene expression data was generated using the HTG EdgeSeq DLBCL Cell of Origin Assay IT for Ion Torrent platform (Ion GeneStudio S5 prime, Thermo Fisher Scientific, Waltham, MA, USA). Starting material was tissue sections using 5 µm formalin-fixed paraffin embedded (FFPE) sections according to the manufactures protocol. Target capture, final libraries and semiconductor sequencing were performed with KAPA Library Quantification Kit, the Ion 510 & Ion 520 & Ion 530 Kit – Chef and the Ion 520 Chip Kit (Thermo Fisher Scientific, Waltham, MA, USA) according to the HTG recommendations, prepared for sequencing for Ion Torrent platform (F. Hoffman - La Roche AG, Basel/Kaiseraugst, Schweiz). The HTG EdgeSeq DLBCL panel measures the expression of 92 genes associated to B-cell lymphomas. The COO classification was performed using the HTG Edge System software (Version 5.5.823.5747). The HTG algorithm is trained to minimise the unclassifiable subgroup when compared with other methods. [1]

## Mutational analysis – targeted Next Generation Sequencing

Targeted mutation analysis was performed by Next Generation Sequencing (Ion GeneStudio S5 prime, Thermo Fisher Scientific) using an AmpliSeq Custom Panel designed for DLBCLs (supplemental Table 1). Amplicon library preparation and semiconductor sequencing was done according to the manufacturers' manuals using the Ion AmpliSeq Library Kit v2.0, the Ion Library TaqMan Quantitation Kit, the Ion 510 & Ion 520 & Ion 530 Kit – Chef, the Ion 520 Chip Kit and the Ion 530 Chip Kit (Thermo Fisher Scientific) as described before [2, 3].

DNA extracted from FFPE material (Maxwell® RSC DNA FFPE Kit and the Maxwell® RSC Instrument (Promega, Madison, WI, USA)) was quantified using the Invitrogen Qubit 3 fluorometer (Thermo Fisher Scientific), according to the manufacturer's protocol. For each sample, two PCR reactions were prepared, using 10 ng of DNA, AmpliSeq HiFi Mix Plus (Thermo Fisher Scientific) and one of the two panel-specific primer pools, containing 95 or 98 amplicons. The PCR run was set up according to the manufacturer's protocol: initial denaturation at 99 °C for 2 minutes, followed by 24 cycles of 99 °C for 15 seconds and 60 °C for 4 minutes. After completing the PCR reaction, an enzymatic partial digestion of the PCR primer was performed using FuPa exonuclease (Thermo Fisher Scientific), followed by an enzymatic ligation of Ion Xpress Barcode Adapters (Thermo Fisher Scientific). The prepared libraries were cleaned up using the HighPrep PCR clean-up system (MagBio Genomics Inc.,

Gaithersburg, MD, USA) and quantified using the Ion Library TaqMan Quantitation Kit (Thermo Fisher Scientific) on a LightCycler 480 qPCR instrument (Roche Molecular Systems). Libraries were pooled and processed to clonal library amplification and enrichment on Ion Sphere particles using the Ion Chef platform (Thermo Fisher Scientific). Sequencing was performed on a 520 or 530 chip on the Ion GeneStudio S5 prime (Thermo Fisher Scientific). The data analysis was performed on the Ion Torrent Software and the Ion Reporter Software (both Thermo Fisher Scientific). The threshold for mutations was set to an allele frequency (VAF) of 5% (6% and 7% in one case each due to the limited integrity of the DNA in these cases). Variant calling of non-synonymous somatic variants compared to the human reference sequence hg19 was performed using Ion Reporter Software (Thermo Fisher Scientific, Version 5.10.1 to Version 5.16.0.2). Variants called by the Ion Reporter Software were visualized using the Integrative Genomics Viewer (IGV; Broad Institute, Cambridge, MA; Version 2.8.0) to exclude panel-specific artefacts. All mutations were verified using the Catalogue of Somatic Mutations in Cancer (COSMIC; Wellcome Sanger Institute, Cambridgeshire, UK), and the reference SNP (rs) report (U.S. National Library of Medicine). Precition scores were created using COSMIC FATHMM, Varsome and CADD.

Not entirely clear assessable variants were validated using the Ion Amplicon Library Preparation Fusion Method (Thermo Fisher Scientific) according to the manufacturers protocol. Primers were designed using the primer3 software (supplemental Table 2).

# Supplemental tables

**Supplemental table 1.** AmpliSeq Custom Panel for the analysis of DLBCL.

| Gene | Transcript | Position (GRCh37/hg19) | Exon(s) | Amplicons* | Coverage of CDS (%) |
|---|---|---|---|---|---|
| ***EZH2*** | NM_004456 | chr7:148,508,712 - chr7:148,508,789 | 16 | 1 | - |
| ***CD79B*** | NM_001039933 | chr17:62,006,789 - chr17:62,006,840 | 5 | 1 | - |
| | | chr17:62,006,586 - chr17:62,006,654 | 6 | 1 | - |
| ***MYD88*** | NM_002468 | chr3:38,181,874 - chr3:38,182,064 | 3 | 4 | - |
| | | chr3:38,182,243 - chr3:38,182,344 | 4 | 2 | - |
| | | chr3:38,182,618 - chr3:38,182,726 | 5 | 2 | - |
| ***CARD11*** | NM_032415 | chr7:2,946,272 - chr7:2,998,140 | CDS | 54 | 95,30 |
| ***IRF4*** | NM_002460 | chr6:393,153 - chr6:407,598 | CDS | 18 | 95,61 |
| ***BCL2*** | NM_000633 | chr18:60,795,858 - chr18:60,985,899 | CDS | 9 | 95,86 |
| ***TNFAIP3*** | NM_006290 | chr6:138,192,365 - chr6:138,202,456 | CDS | 29 | 96,49 |
| ***PRDM1*** | NM_001198 | chr6:106,534,429 - chr6:106,555,361 | CDS | 29 | 91,66 |
| ***BCL6*** | NM_001706 | chr3:187,440,246 - chr3:187,451,481 | CDS | 27 | 92,14 |
| ***PIM1*** | NM_002648 | chr6:37,138,352 - chr6:37,141,867 | CDS | 16 | 90,98 |

* The amplicon lengths range between 125-175 bp.

CDS: coding sequence. Coverage of the panel in total: 94,23%. Amplicons in total: 193. Amplicons pool 1: 98. Amplicons pool 2: 95.

**Supplemental table 2.** Custom single amplicons for the NGS targeted sequencing. Primer sequences for targeted resequencing using the Ion Amplicon Library Preparation Fusion Method including the sequences of the A or trP1 adapter and the barcode sequence.

| Gene | Primer name | Primer sequence (5´-3´) |
|---|---|---|
| ***EZH2*** | EZH2 Ex16 646 BC63 AF | CCATCTCATCCCTGCGTGTCTCCGACTCAGCCTTAGAGTTCGATTATTGCTGGCACCATCTGAC |
| | EZH2 Ex16 646 BC63 AR | CCATCTCATCCCTGCGTGTCTCCGACTCAGCCTTAGAGTTCGATTGAATACAGGTTATCAGTGCCTT |
| | EZH2 Ex16 646 trP1F | CCTCTCTATGGGCAGTCGGTGATTATTGCTGGCACCATCTGAC |
| | EZH2 Ex16 646 trP1R | CCTCTCTATGGGCAGTCGGTGATTGAATACAGGTTATCAGTGCCTT |
| | EZH2 Ex16 646 BC64 AF | CCATCTCATCCCTGCGTGTCTCCGACTCAGCTGAGTTCCGACGATTATTGCTGGCACCATCTGAC |
| | EZH2 Ex16 646 BC64 AR | CCATCTCATCCCTGCGTGTCTCCGACTCAGCTGAGTTCCGACGATTGAATACAGGTTATCAGTGCCTT |
| | EZH2 Ex16 646 trP1F | CCTCTCTATGGGCAGTCGGTGATTATTGCTGGCACCATCTGAC |
| | EZH2 Ex16 646 trP1R | CCTCTCTATGGGCAGTCGGTGATTGAATACAGGTTATCAGTGCCTT |
| | EZH2 Ex16 646 BC65 AF | CCATCTCATCCCTGCGTGTCTCCGACTCAGTCCTGGCACATCGATTATTGCTGGCACCATCTGAC |
| | EZH2 Ex16 646 BC65 AR | CCATCTCATCCCTGCGTGTCTCCGACTCAGTCCTGGCACATCGATTGAATACAGGTTATCAGTGCCTT |
| | EZH2 Ex16 646 trP1F | CCTCTCTATGGGCAGTCGGTGATTATTGCTGGCACCATCTGAC |
| | EZH2 Ex16 646 trP1R | CCTCTCTATGGGCAGTCGGTGATTGAATACAGGTTATCAGTGCCTT |
| ***IRF4*** | IRF4_Ex2_BC91A_F | CCATCTCATCCCTGCGTGTCTCCGACTCAGCGGAAGGATGCGATCGGGGCATGAACCTGGAG |
| | IRF4_Ex2_BC91A_R | CCATCTCATCCCTGCGTGTCTCCGACTCAGCGGAAGGATGCGATCGGTTGTAGTCCTGCTTGC |
| | IRF4_Ex2_trP1_F | CCTCTCTATGGGCAGTCGGTGATCGGGGCATGAACCTGGAG |
| | IRF4_Ex2_trP1_R | CCTCTCTATGGGCAGTCGGTGATCGGTTGTAGTCCTGCTTGC |
| | IRF4_A1-35_BC69A_F | CCATCTCATCCCTGCGTGTCTCCGACTCAGTTCAATTGGCGATTCTCCCCGCAGTGCAGAG |
| | IRF4_A1-35_BC69A_R | CCATCTCATCCCTGCGTGTCTCCGACTCAGTTCAATTGGCGATTCGTTCTCCCACACCAGC |
| | IRF4_A1-35_trP1_F | CCTCTCTATGGGCAGTCGGTGATTCTCCCCGCAGTGCAGAG |
| | IRF4_A1-35_trP1_R | CCTCTCTATGGGCAGTCGGTGATTCGTTCTCCCACACCAGC |
| | IRF4_35-72_BC69A_F | CCATCTCATCCCTGCGTGTCTCCGACTCAGTTCAATTGGCGATGAAGCTCCGCCAGTGG |
| | IRF4_35-72_BC69A_R | CCATCTCATCCCTGCGTGTCTCCGACTCAGTTCAATTGGCGATCTCTGTCTCTGGGCCCTC |

| Gene | Primer name | Primer sequence (5´-3´) |
|---|---|---|
| | IRF4_35-72_trP1_F | CCTCTCTATGGGCAGTCGGTGATGAAGCTCCGCCAGTGG |
| | IRF4_35-72_trP1_R | CCTCTCTATGGGCAGTCGGTGATCTCTGTCTCTGGGCCCTC |
| ***CARD11*** | CARD11 Ex20 871 BC58 AF | CCATCTCATCCCTGCGTGTCTCCGACTCAGTCCTAGAACACGATAGGGCCTGACTGATTGATAAAT |
| | CARD11 Ex20 871 BC58 AR | CCATCTCATCCCTGCGTGTCTCCGACTCAGTCCTAGAACACGATCTGAAGGAGCTGGCCAAAA |
| | CARD11 Ex20 871 trP1F | CCTCTCTATGGGCAGTCGGTGATAGGGCCTGACTGATTGATAAAT |
| | CARD11 Ex20 871 trP1R | CCTCTCTATGGGCAGTCGGTGATCTGAAGGAGCTGGCCAAAA |
| | CARD11 Ex23/24 1046 BC60 AF | CCATCTCATCCCTGCGTGTCTCCGACTCAGTCTAGCTCTTCGATCTCAGAAGGCAGAAGACGGA |
| | CARD11 Ex23/24 1046 BC60 AR | CCATCTCATCCCTGCGTGTCTCCGACTCAGTCTAGCTCTTCGATCATCCAACCTCCCAGTCCC |
| | CARD11 Ex23/24 1046 trP1F | CCTCTCTATGGGCAGTCGGTGATCTCAGAAGGCAGAAGACGGA |
| | CARD11 Ex23/24 1046 trP1R | CCTCTCTATGGGCAGTCGGTGATCATCCAACCTCCCAGTCCC |
| | CARD11_Ex5_BC33A_F | CCATCTCATCCCTGCGTGTCTCCGACTCAGTTCTCATTGAACGATTGGAGCTGCTAACCTTCCAG |
| | CARD11_Ex5_BC33A_R | CCATCTCATCCCTGCGTGTCTCCGACTCAGTTCTCATTGAACGATGTCTCGGCTCCTCATGACC |
| | CARD11_Ex5_trP1_F | CCTCTCTATGGGCAGTCGGTGATTGGAGCTGCTAACCTTCCAG |
| | CARD11_Ex5_trP1_R | CCTCTCTATGGGCAGTCGGTGATGTCTCGGCTCCTCATGACC |
| ***PIM1*** | PIM1_Ex4_124_BC71_F | CCATCTCATCCCTGCGTGTCTCCGACTCAGTGAGGCTCCGACGATAAGAAGGTGAGCTCGGGTTT |
| | PIM1_Ex4_124_BC71_R | CCATCTCATCCCTGCGTGTCTCCGACTCAGTGAGGCTCCGACGATTTTTCGTCCTTGATGTCGCG |
| | PIM1_Ex4_124_trP1_F | CCTCTCTATGGGCAGTCGGTGATAAGAAGGTGAGCTCGGGTTT |
| | PIM1_Ex4_124_trP1_R | CCTCTCTATGGGCAGTCGGTGATTTTTCGTCCTTGATGTCGCG |
| ***BCL2*** | BCL2_Ex2_192_BC71_F | CCATCTCATCCCTGCGTGTCTCCGACTCAGTGAGGCTCCGACGATGCCTTCTTTGAGTTCGGTGG |
| | BCL2_Ex2_192_BC71_R | CCATCTCATCCCTGCGTGTCTCCGACTCAGTGAGGCTCCGACGATCACCAAGTGCACCTACCCA |
| | BCL2_Ex2_192_trP1_F | CCTCTCTATGGGCAGTCGGTGATGCCTTCTTTGAGTTCGGTGG |
| | BCL2_Ex2_192_trP1_R | CCTCTCTATGGGCAGTCGGTGATCACCAAGTGCACCTACCCA |

**Supplemental table 3.** Overview of the NGS analyses performed.

| Case | Panel Analysis | Single Amplicons |
|---|---|---|
| **1** | AmpliSeq Custom DLBCL Panel | EZH2 Ex16 646<br>IRF4 Ex2 35-72 |
| **2** | AmpliSeq Custom DLBCL Panel | CARD11 Ex20 871<br>EZH2 Ex16 646<br>IRF4 Ex2 35-72 |
| **5** | AmpliSeq Custom DLBCL Panel | EZH2 Ex16 646<br>IRF4 Ex2 35-72 |
| **6** | AmpliSeq Custom DLBCL Panel | IRF4 Ex2 35-72<br>EZH2 Ex16 646 |
| **7** | AmpliSeq Custom DLBCL Panel | IRF4 Ex2 35-72 |
| **8** | AmpliSeq Custom DLBCL Panel | IRF4 Ex2 35-72 |
| **9** | AmpliSeq Custom DLBCL Panel | CARD11 Ex5 215<br>IRF4 Ex2 35-72 |
| **10** | AmpliSeq Custom DLBCL Panel | IRF4 Ex2 35-72 |
| **11** | AmpliSeq Custom DLBCL Panel | CARD11 Ex23 1046<br>IRF4 Ex2 35-72 |
| **13** | AmpliSeq Custom DLBCL Panel | CARD11 Ex23 1046<br>IRF4 Ex2 1-35<br>IRF4 Ex2 35-72 |
| **15** | AmpliSeq Custom DLBCL Panel | CARD11 Ex23 1046<br>EZH2 Ex16 646<br>IRF4 Ex2 35-72 |
| **16** | AmpliSeq Custom DLBCL Panel | CARD11 Ex23 1046<br>IRF4 Ex2 35-72 |
| **17** | AmpliSeq Custom DLBCL Panel | CARD11 Ex23 1046<br>IRF4 Ex2 35-72 |
| **19** | AmpliSeq Custom DLBCL Panel | EZH2 Ex16 646<br>IRF4 Ex2 35-72 |
| **20** | AmpliSeq Custom DLBCL Panel | BCL2 Ex2 53<br>IRF4 Ex2 35-72<br>EZH2 Ex16 646 |
| **21** | AmpliSeq Custom DLBCL Panel | IRF4 Ex2 35-72 |

| Case | Panel Analysis | Single Amplicons |
|---|---|---|
| | | EZH2 Ex16 646 |
| **22** | AmpliSeq Custom DLBCL Panel | IRF4 Ex2 35-72 |
| **23** | AmpliSeq Custom DLBCL Panel | IRF4 Ex2 35-72 |
| **24** | AmpliSeq Custom DLBCL Panel | IRF4 Ex2 35-72 |
| **25** | AmpliSeq Custom DLBCL Panel | EZH2 Ex16 646<br>IRF4 Ex2 35-72 |
| **26** | AmpliSeq Custom DLBCL Panel | IRF4 Ex2 35-72<br>BCL2 Ex2 192 |
| **27** | AmpliSeq Custom DLBCL Panel | IRF4 Ex2 35-72 |
| **29** | AmpliSeq Custom DLBCL Panel | EZH2 Ex16 646<br>IRF4 Ex2 35-72 |
| **30** | AmpliSeq Custom DLBCL Panel | IRF4 Ex2 35-72 |
| **31** | AmpliSeq Custom DLBCL Panel | IRF4 Ex2 35-72 |
| **32** | AmpliSeq Custom DLBCL Panel | EZH2 Ex16 646<br>IRF4 Ex2 35-72 |
| **33** | AmpliSeq Custom DLBCL Panel | EZH2 Ex16 646<br>IRF4 Ex2 35-72<br>PIM1 Ex4 124 |
| **34** | AmpliSeq Custom DLBCL Panel | IRF4 Ex2 35-72 |
| **35** | AmpliSeq Custom DLBCL Panel | IRF4 Ex2 1-35<br>IRF4 Ex2 35-72 |
| **36** | AmpliSeq Custom DLBCL Panel | IRF4 Ex2 1-35<br>IRF4 Ex2 35-72 |

64

**Supplemental table 4.** List of all mutations detected.

| # | VAF Thres hold | Gene | Transcript | Coordinates | VAF | Coverage | cDNA change | Protein change | Prediction | | |
|---|---|---|---|---|---|---|---|---|---|---|---|
| | | | | | | | | | COSMIC FATHMM | Varsome | CADD |
| **2** | 5% | *CD79B* | NM_001039933 | chr17:62006798 | 29% | 2076 | c.590A>C | p.Y197S | Pathogenic (score 0.94) | Likely pathogenic | 24.5 |
| **5** | 5% | *TNFAIP3* | NM_001270507 | chr6:138198338 | 10% | 10385 | c.931G>T | p.E311* | – | Pathogenic | 38 |
| **8** | 5% | *BCL2* | NM_000633 | chr18:60985692 | 45% | 2488 | c.208T>G | p.S70A | – | Uncertain significance | 17.07 |
| | | *EZH2* | NM_004456 | chr7:148508728 | 14% | 190 | c.1936T>A | p.Y646N | Pathogenic (score 0.99) | Likely pathogenic | 32 |
| **9** | 5% | *PIM1* | NM_002648 | chr6:37139097 | 19% | 33595 | c.437G>A | p.S146N | Pathogenic (score 0.78) | Uncertain significance | 21.9 |
| | | *CARD11* | NM_032415 | chr7:2983885 | 10% | 5299 | c.645G>C | p.K215N | – | Uncertain significance | 22 |
| **10** | 5% | *MYD88* | NM_002468 | chr3:38182292 | 50% | 18078 | c.728G>A | p.S243N | – | Pathogenic | 28.7 |
| | | *PIM1* | NM_002648 | chr6:37138354 | 14% | 3582 | c.3G>A | p.M1I | – | Pathogenic | 24.1 |
| | | *PIM1* | NM_002648 | chr6:37138423 | 13% | 3699 | c.72G>C | p.K24N | – | Uncertain significance | 18.57 |
| **11** | 5% | *PRDM1* | NM_001198 | chr6:106536223 - chr6:106536233 | 50% | 34216 | c.190_200dup | p.D68Tfs | – | Pathogenic | N/A |
| **13** | 5% | *PRDM1* | NM_001198 | chr6:106547183 | 51% | 7353 | c.420delC | p.R141Efs | – | Pathogenic | 31 |
| | | *IRF4* | NM_002460 | chr6:393187 | 23% | 1217 | c.35T>G | p.F12C | – | Uncertain significance | 23.3 |

| # | VAF Thres hold | Gene | Transcript | Coordinates | VAF | Coverage | cDNA change | Protein change | Prediction | | |
|---|---|---|---|---|---|---|---|---|---|---|---|
| | | | | | | | | | COSMIC FATHMM | Varsome | CADD |
| | | *IRF4* | NM_002460 | chr6:393190 | 23% | 1218 | c.38G>C | p.G13A | – | Uncertain significance | 25 |
| | | *IRF4* | NM_002460 | chr6:393208 | 22% | 3827 | c.56G>T | p.C19F | – | Uncertain significance | 28.9 |
| | | *IRF4* | NM_002460 | chr6:393252 | 28% † | 61399 | c.100A>G | p.S34G | – | Uncertain significance | 27 |
| | | *IRF4* | NM_002460 | chr6:393260 | 42% † | 61720 | c.108G>T | p.K36N | Pathogenic (score 0.91) | Uncertain significance | 24.8 |
| | | *IRF4* | NM_002460 | chr6:393295 | 26% † | 64929 | c.143G>A | p.S48N | – | Uncertain significance | 25 |
| | | *IRF4* | NM_002460 | chr6:393332 | 38% † | 65135 | c.180G>C | p.Q60H | Pathogenic (score 0.87) | Uncertain significance | 24.9 |
| | | *IRF4* | NM_002460 | chr6:393342 | 41% † | 65920 | c.190C>T | p.R64C | – | Uncertain significance | 32 |
| **15** | 5% | *PIM1* | NM_002648 | chr6:37139062 - chr6:37139070 | 89% | 4100 | c.402_410del | p.E135_G137del | – | Uncertain significance | N/A |
| | | *CD79B* | NM_001039933 | chr17:62006798 | 42% | 4652 | c.590A>T | p.Y197F | Pathogenic (score 0.94) | Uncertain significance | 24 |
| | | *BCL2* | NM_000633 | chr18:60985889 | 14% | 4434 | c.11C>T | p.A4V | Pathogenic (score 0.92) | Uncertain significance | 22.7 |
| **17** | 5% | *BCL2* | NM_000633 | chr18:60985743 | 89% | 2300 | c.157C>T | p.P53S | Neutral (score 0.04) | Uncertain significance | 11.12 |
| | | *BCL2* | NM_000633 | chr18:60985594 | 58% | 5600 | c.306C>A | p.D102E | Neutral (score 0.01) | Uncertain significance | 21.2 |

| # | VAF Thres hold | Gene | Transcript | Coordinates | VAF | Coverage | cDNA change | Protein change | Prediction | | |
|---|---|---|---|---|---|---|---|---|---|---|---|
| | | | | | | | | | COSMIC FATHMM | Varsome | CADD |
| | | *BCL2* | NM_000633 | chr18:60985590 | 58% | 5600 | c.310T>A | p.F104I | – | Uncertain significance | 22.2 |
| | | *CARD11* | NM_032415 | chr7:2977614 | 51% | 11300 | c.1070A>T | p.D357V | Pathogenic (score 0.94) | Uncertain significance | 28.2 |
| | | *CARD11* | NM_032415 | chr7:2979453 | 46% | 3970 | c.794A>C | p.Q265P | – | Uncertain significance | 21.6 |
| | | *BCL2* | NM_000633 | chr18:60985890 | 45% | 2300 | c.10G>C | p.A4P | Neutral (score 0.44) | Uncertain significance | 22.2 |
| | | *BCL2* | NM_000633 | chr18:60985886 | 45% | 2300 | c.14G>A | p.G5E | – | Uncertain significance | 25.3 |
| | | *PIM1* | NM_002648 | chr6:37139210 | 36% | 13990 | c.550C>G | p.L184V | Pathogenic (score 0.94) | Uncertain significance | 27.3 |
| **19** | 5% | *MYD88* | NM_002468 | chr3:38182641 | 84% | 5946 | c.794T>C | p.L265P | Pathogenic (score 0.96) | Pathogenic | 32 |
| | | *PRDM1* | NM_001198 | chr6:106534471 | 61% | 837 | c.42+1G>C | p.? | – | Pathogenic | 35 |
| | | *PRDM1* | NM_001198 | chr6:106554273 | 59% | 2282 | c.1801C>T | p.R601W | – | Uncertain significance | 32 |
| | | *PIM1* | NM_002648 | chr6:37138802 - chr6:37138804 | 38% | 3128 | c.235_237delinsAAA | p.E79K | Pathogenic (score 0.97) | Uncertain significance | 23.4 |
| **20** | 7% | *MYD88* | NM_002468 | chr3:38182641 | 52% | 9706 | c.794T>C | p.L265P | Pathogenic (score 0.96) | Pathogenic | 32 |
| **21** | 5% | *BCL2* | NM_000633 | chr18:60985840 | 33% | 5426 | c.60T>G | p.H20Q | N/A | Uncertain significance | 21.9 |

| # | VAF Thres hold | Gene | Transcript | Coordinates | VAF | Coverage | cDNA change | Protein change | Prediction | | |
|---|---|---|---|---|---|---|---|---|---|---|---|
| | | | | | | | | | COSMIC FATHMM | Varsome | CADD |
| | | *CARD11* | NM_032415 | chr7:2977614 | 30% | 8364 | c.1070A>T | p.D357V | Pathogenic (score 0.94) | Uncertain significance | 29.8 |
| | | *BCL6* | NM_001706 | chr3:187444538 | 30% | 10326 | c.1689C>G | p.S563R | – | Uncertain significance | 28.8 |
| **23** | 5% | *MYD88* | NM_002468 | chr3:38182032 | 28% | 11828 | c.656C>G | p.S219C | Pathogenic (score 0.96) | Uncertain significance | 29.1 |
| **24** | 5% | *PIM1* | NM_002648 | chr6:37138769 | 35% | 1057 | c.202C>T | p.H68Y | Pathogenic (score 0,97) | Uncertain significance | 22.4 |
| | | *BCL2* | NM_000633 | chr18:60985549 | 7% | 2238 | c.351C>G | p.S117R | Neutral (score 0,03) | Uncertain significance | 14.26 |
| | | *BCL2* | NM_000633 | chr18:60985562 | 8% | 2211 | c.338C>T | p.A113V | Neutral (score 0,03) | Uncertain significance | 14.07 |
| | | *BCL2* | NM_000633 | chr18:60985575 | 8% | 2216 | c.325C>G | p.R109G | – | Uncertain significance | 24.7 |
| | | *BCL2* | NM_000633 | chr18:60985635 | 32% | 658 | c.265G>A | p.V89M | – | Uncertain significance | 22.4 |
| | | *BCL2* | NM_000633 | chr18:60985644 | 32% | 644 | c.256C>G | p.L86V | Neutral (score 0,24) | Uncertain significance | 17.47 |
| **25** | 5% | *CARD11* | NM_032415 | chr7:2958135 | 55% | 347 | c.2597G>A | p.R866Q | Neutral (score 0.22) | Uncertain significance | 12.47 |
| **26** | 6% | *PIM1* | NM_002648 | chr6:37138365 | 35% | 1027 | c.14A>G | p.K5R | – | Uncertain significance | 24.1 |
| | | *PIM1* | NM_002648 | chr6:37138367 | 35% | 1027 | c.16A>G | p.I6V | – | Uncertain significance | 18.74 |

| # | VAF Threshold | Gene | Transcript | Coordinates | VAF | Coverage | cDNA change | Protein change | Prediction | | |
|---|---|---|---|---|---|---|---|---|---|---|---|
| | | | | | | | | | COSMIC FATHMM | Varsome | CADD |
| | | *PIM1* | NM_002648 | chr6:37138901 | 30% | 2088 | c.241C>T | p.P81S | Pathogenic (score 0,97) | Uncertain significance | 22.8 |
| | | *EZH2* | NM_004456 | chr7:148508727 | 30% ‡ | 5985 | c.1937A>G | p.Y646C | Pathogenic (score 0,99) | Likely pathogenic | 25.2 |
| | | *BCL2* | NM_000633 | chr18:60985644 | 34% | 1002 | c.256C>T | p.L86F | Neutral (score 0,29) | Uncertain significance | 19.64 |
| | | *BCL2* | NM_000633 | chr18:60985326 | 9% ‡ | 256782 | c.574A>C | p.N192H | Neutral (0,10) | Uncertain significance | 28,2 |
| **27** | 5% | *BCL2* | NM_000633 | chr18:60795869 | 30% | 9448 | c.709G>A | p.G237S | – | Uncertain significance | 24.5 |
| | | *BCL2* | NM_000633 | chr18:60985840 | 24% | 4187 | c.60T>A | p.H20Q | N/A | Uncertain significance | 21.9 |
| **31** | 5% | *CD79B* | NM_001039933 | chr17:62006793 | 62% | 9807 | c.594+1G>C | p.? | – | Pathogenic | 32 |
| **33** | 5% | *PIM1* | NM_002648 | chr6:37139078 | 13% ‡ | 152732 | c.418C>T | p.Q140* | N/A | Pathogenic | 37 |
| | | *PIM1* | NM_002648 | chr6:37139063 | 11% ‡ | 152732 | c.403G>T | p.E135* | – | Pathogenic | 37 |
| **35** | 5% | *IRF4* | NM_002460 | chr6:393172 | 31% | 903 | c.20G>T | p.G7V | – | Uncertain significance | 22 |
| | | *IRF4* | NM_002460 | chr6:393201 | 11% | 911 | c.49G>C | p.V17L | – | Uncertain significance | 22.6 |
| | | *IRF4* | NM_002460 | chr6:393206 | 11% | 906 | c.54C>A | p.S18R | – | Uncertain significance | 22.6 |
| | | *IRF4* | NM_002460 | chr6:393262 | 21% † | 2285 | c.110A>T | p.Y37F | – | Uncertain significance | 28.4 |

| # | VAF Threshold | Gene | Transcript | Coordinates | VAF | Coverage | cDNA change | Protein change | Prediction | | |
|---|---|---|---|---|---|---|---|---|---|---|---|
| | | | | | | | | | COSMIC FATHMM | Varsome | CADD |
| | | *IRF4* | NM_002460 | chr6:393283 | 21% † | 2444 | c.131A>G | p.N44S | – | Uncertain significance | 27 |
| | | *IRF4* | NM_002460 | chr6:393295 | 16% † | 2310 | c.143G>C | p.S48T | – | Uncertain significance | 21.1 |
| | | *IRF4* | NM_002460 | chr6:393298 | 23% † | 2496 | c.146T>C | p.I49T | – | Uncertain significance | 25.4 |
| | | *IRF4* | NM_002460 | chr6:393330 - chr6:393332 | 23% † | 2575 | c.178_180delinsTTC | p.Q60F | – | Uncertain significance | N/A |

† Covered only in the single amplicon, frequency therefore of the single amplicon.

‡ Covered in both single amplicon and panel, frequency of single amplicon.

# Supplemental figures

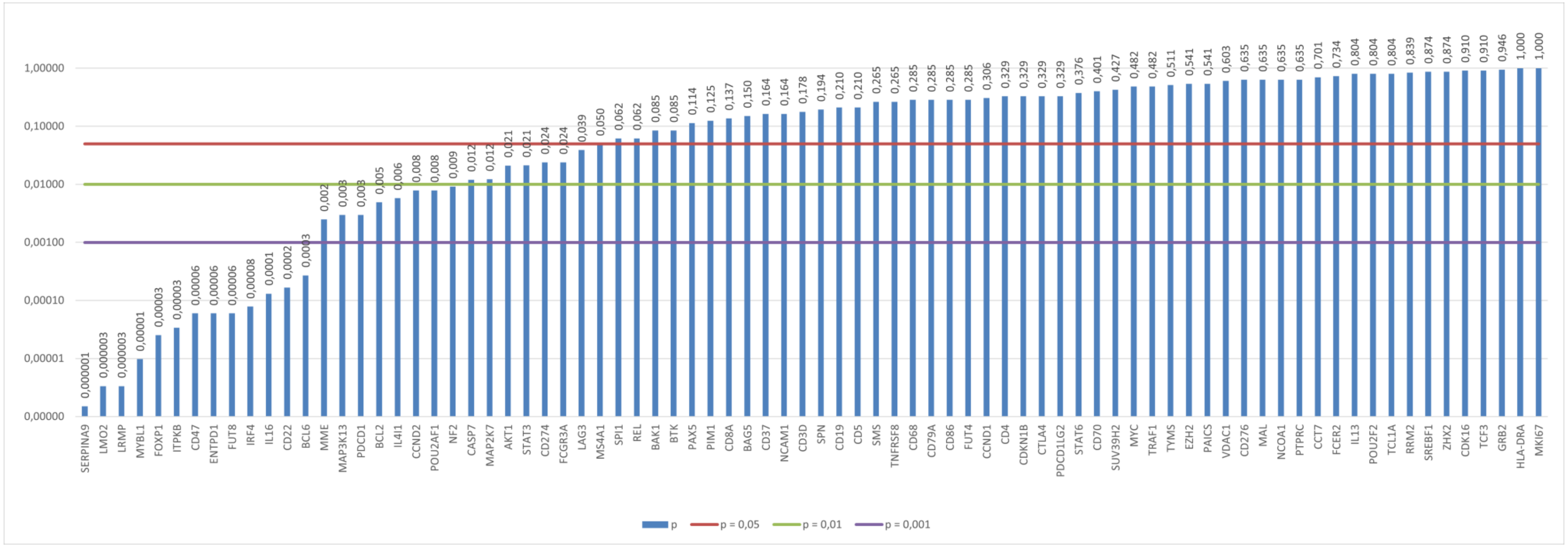


**Supplemental Fig. 1: p values of Mann Whitney U test of the distribution of gene expression levels across the GEP subtype (GCB – ABC) (log10 y-Axis).** Each bar on the x-axis represents the p-value of one statistical test. The red, green, and purple horizontal lines represent the significance levels of p = .05, p = .005 and p = .001. At the significance level p ≤ 0.001, a total of 13 of the genes examined resp. the corresponding mRNAs showed significant differences in gene expression between the subgroups. For the significance level p ≤ 0.01, this was the case for a total of 21 of the genes resp. mRNAs examined (including the 13 genes resp. mRNAs significant at p ≤ 0.001), for p ≤ 0.05 for a total of 29 genes resp. mRNAs.

(Supplemental Figure 1 was created using Microsoft Excel version 2210.)

# Supplemental references

1. Ahmed S, Glover P, Taylor J, Sha C, Care MA, Tooze R, Davies A, Westhead DR, Johnson PWM, Burton C, Barrans SL (2021) Comparative analysis of gene expression platforms for cell-of-origin classification of diffuse large B-cell lymphoma shows high concordance British Journal of Haematology 192:599-604. doi: 10.1111/bjh.17246

2. Nann D, Ramis-Zaldivar JE, Müller I, Gonzalez-Farre B, Schmidt J, Egan C, Salmeron-Villalobos J, Clot G, Mattern S, Otto F, Mankel B, Colomer D, Balagué O, Szablewski V, Lome-Maldonado C, Leoncini L, Dojcinov S, Chott A, Copie-Bergman C, Bonzheim I, Fend F, Jaffe ES, Campo E, Salaverria I, Quintanilla-Martinez L (2020) Follicular lymphoma t(14;18)-negative is genetically a heterogeneous disease Blood Advances 4:5652-5665. doi: 10.1182/bloodadvances.2020002944

3. Frauenfeld L, Castrejon-de-Anta N, Ramis-Zaldivar JE, Streich S, Salmerón-Villalobos J, Otto F, Mayer AK, Steinhilber J, Pinyol M, Mankel B, Ramsower C, Bonzheim I, Fend F, Rimsza LM, Salaverria I, Campo E, Balagué O, Quintanilla-Martinez L (2022) Diffuse large B-cell lymphomas in adults with aberrant coexpression of CD10, BCL6, and MUM1 are enriched in IRF4 rearrangements Blood Advances 6:2361-2372. doi: 10.1182/bloodadvances.2021006034
